# Case of Idiopathic Phlebitis

**Published:** 1831

**Authors:** 


### Case of Idiopathic Phlebitis. By Paul S. Knight, M.D.

The following case is related in the second number of the North of England Medical and Surgical Journal, by Dr. Knight, of Glossop.

"Joseph Harrop, aged forty-eight, is a strong muscular man, but considerably the worse from wear and tear. He served in the Peninsular wars in the Horse Artillery, and was there wounded by musket shot, once on the lower part of the right tibia, and another time the ball penetrated through the inner part of the thigh, passing pretty near the trochanter minor, also on the right side; he also suffered much from exposure at nights, and excessive fatigue, and he supposes that, in consequence, the veins of both legs have become varicose. In November last he was employed as a watchman, and during one very cold night he suffered much, and shortly after pain and erysipelatous inflammation made its appearance on the inner side of the left leg up the course of the absorbents; and for which he took mercurial purgatives, and the carbon. sodæ, and applied to the part leeches and a sedative lotion. On the 2nd December, about a fortnight after the first application for relief, the man complained of headach, with a pulse about 90 and sharp, and a countenance expressive of great anxiety; I found the vena saphena inflamed, and to the touch it conveyed the sensation of a cord under the integuments, extending from a little above the inner condyle of the knee, up to the abdominal ring, the integuments of the knee immediately above the vena saphena in a state of very active inflammation, and the vein itself, at that part, perceptible to the eye, bulging and large, and somewhat elastic on pressure. As this tumour produced extreme pain, *and as it was quite clear that all circulating communication was cut off*, I resolved to open it freely, a large clot of coagulated blood instantly plugged up the orifice but was gradually protruded out, and clot after clot succeeded, great ease being the speedy result. Below this spot I was subsequently obliged to open two abscesses that had formed in the vein, and from which healthy pus mixed with coagulated venous blood was discharged; these abscesses healed readily, but repeated local and general bleeding, the latter performed in the erect posture, and continued till fainting was produced, was necessary to subdue the soreness of the part. This had scarcely been accomplished on the 19th Dec. 1829, when the poor fellow directed my attention to the vena saphena of the right thigh, it was inflamed from below the knee up to the abdominal ring, that is, it felt extremely sore, and produced to the touch the same impression that a cord would, a slight blush was visible immediately over the vein—thirty-six ounces of blood were taken from the arm, and two dozen leeches applied along the course of the inflamed vein.—℞. Pil. Hydrarg. ℈i. in bol. ii. divide capiat i. nocte maneque.

20th Dec.—Much pain on pressure—pulse hard and full, countenance very copious, complains of headach, bowels open.

V. S. ad ℔ii. and apply twenty-four more leeches.—℞. Subm. Hydrarg. ʒi. Opii. purif. gr. iv. Cons. Ros. q. s. ut. ft. massa in pilulas xxiv. dividend. quarum capiat unam quarta quaque hora.

21st.—Blood taken yesterday much cupped and buff colored. Pulse 80, and sharp—continue the pills. Pulse softer.

22nd.—Mouth affected, pulse soft and free, a hard tumour in the groin, as felt yesterday, is separated into detached and small hard masses; the ptyalism was continued about a fortnight, when all inflammatory action had subsided."

